# Cardiac Evaluation Using Two-Dimensional Speckle-Tracking Echocardiography and Conventional Echocardiography in Taiwanese Patients with Mucopolysaccharidoses

**DOI:** 10.3390/diagnostics10020062

**Published:** 2020-01-23

**Authors:** Hsiang-Yu Lin, Chih-Kuang Chuang, Chung-Lin Lee, Ming-Ren Chen, Kuo-Tzu Sung, Shan-Miao Lin, Charles Jia-Yin Hou, Dau-Ming Niu, Tung-Ming Chang, Chung-Lieh Hung, Shuan-Pei Lin

**Affiliations:** 1 Department of Medicine, MacKay Medical College, New Taipei City 25245, Taiwan; lxc46199@ms37.hinet.net (H.-Y.L.); mingren44@gmail.com (M.-R.C.); 8905012@gmail.com (K.-T.S.); miao1029@gmail.com (S.-M.L.); 2 Department of Pediatrics, MacKay Memorial Hospital, Taipei 10449, Taiwan; 3 Department of Medical Research, MacKay Memorial Hospital, New Taipei City 25160, Taiwan; mmhcck@gmail.com; 4 MacKay Junior College of Medicine, Nursing and Management, Taipei 11260, Taiwan; 5 Department of Medical Research, China Medical University Hospital, China Medical University, Taichung 40402, Taiwan; 6 Institute of Biomedical Sciences, MacKay Medical College, New Taipei City 25245, Taiwan; 7 College of Medicine, Fu-Jen Catholic University, Taipei 24205, Taiwan; 8 Department of Pediatrics, MacKay Memorial Hospital, Hsinchu 30071, Taiwan; clampcage@yahoo.com.tw; 9 Institute of Clinical Medicine, National Yang-Ming University, Taipei 11221, Taiwan; dmniu1111@yahoo.com.tw; 10 Division of Cardiology, Department of Internal Medicine, MacKay Memorial Hospital, Taipei 10449, Taiwan; jiayinhou@gmail.com; 11 Department of Pediatrics, Taipei Veterans General Hospital, Taipei 11217, Taiwan; 12 Department of Pediatric Neurology, Changhua Christian Children’s Hospital, Changhua 500, Taiwan; 128658@cch.org.tw; 13 School of Medicine, Kaohsiung Medical University, Kaohsiung 80708, Taiwan; 14 Department of Infant and Child Care, National Taipei University of Nursing and Health Sciences, Taipei 11219, Taiwan

**Keywords:** cardiac, conventional echocardiography, global longitudinal strain, mucopolysaccharidosis, speckle-tracking echocardiography

## Abstract

Background: Mucopolysaccharidoses (MPSs) are a group of rare inherited metabolic disorders that can damage various organs, including the heart. Cardiac abnormalities have been observed in patients with all MPS types, with the most documented abnormalities being cardiac valvular regurgitation and stenosis, valvular thickening, and hypertrophic cardiomyopathy. Methods: Cardiac features of 53 Taiwanese patients with MPS (31 men and 22 women; age range 1.1–34.9 years; seven with MPS I, 16 with MPS II, nine with MPS III, 14 with MPS IVA, and seven with MPS VI) were evaluated using two-dimensional speckle-tracking echocardiography and conventional echocardiography. Results: The mean *z* scores of the global longitudinal strain (GLS), left ventricular mass index (LVMI), interventricular septum diameter in diastole (IVSd), left ventricular posterior wall diameter in diastole (LVPWd), and aortic diameter of the 53 patients with MPS were 1.71, 0.35, 1.66, 1.03, and 3.15, respectively. Furthermore, *z* scores >2 were identified in 45%, 13%, 40%, 13%, and 70% of the GLS, LVMI, IVSd, LVPWd, and aortic diameter, respectively. The most severe GLS was observed in those with MPS VI, followed by in those with MPS II and MPS I. The GLS *z* score was positively correlated with the LVMI *z* score (*p* < 0.01). Moreover, diastolic dysfunction (reversed ratio between early and late (atrial) ventricular filling velocity (E/A ratio < 1)) was identified in 12 patients (23%). Ejection and shortening fractions were abnormal in four (8%) and seven (13%) patients, respectively. Mitral regurgitation (MR) (92%) was the most common valvular heart disease, followed by aortic regurgitation (AR) (57%), mitral stenosis (MS) (21%), and aortic stenosis (AS) (15%). The *z* scores of the GLS and LVMI and severity scores of the MS, MR, AS, and AR were all positively correlated with increasing age (*p* < 0.05). Twenty patients (38%) had a left ventricular remodeling pattern. Conclusions: The most significant left ventricular myocardial deformation, hypertrophy and valvular heart disease were observed in the patients with MPS VI, II, and I, followed by those with MPS IV; in contrast, patients with MPS III had the mildest manifestations. Cardiac abnormalities in patients with MPS worsened with increasing age in accordance with the progressive nature of this disease.

## 1. Introduction

Mucopolysaccharidoses (MPS; OMIM 252700) are a group of rare inherited metabolic disorders resulting from a specific lysosomal enzyme deficiency associated with glycosaminoglycan (GAG) accumulation in various cells and tissues, leading to a progressive multiple organ dysfunction. The development of patients with MPS is usually unaffected at birth; however, multiple clinical manifestations may subsequently appear including coarse facial features, corneal clouding, hearing impairment, skeletal abnormalities, profound growth retardation, poor joint range of motion, hepatosplenomegaly, cardiac hypertrophy, and valvular heart disease. Seven major types of MPS disorders (I, II, III, IV, VI, VII, and IX) consisting of 11 diverse enzyme deficiencies have been identified. All MPS disorders are inherited with an autosomal recessive pattern, except for MPS II, which is an X-linked trait that mainly affects men. Patients with various types of MPS generally have a wide clinical heterogeneity, ranging from attenuated to severe forms in MPS I (Hurler, Hurler-Scheie (H/S), and Scheie syndromes), MPS II, and MPS VI [1,2]. The incidence of MPS in different populations ranges from 1.9 to 4.5 per 100,000 live births. In Taiwan, the collective birth incidence of all MPS types is estimated to be 2.04 per 100,000 live births [3].

Patients with all MPS types suffer from cardiac abnormalities, including cardiac hypertrophy, cardiac valve thickening, and valvular regurgitation and stenosis. The deformed valves with mitral or aortic leaflet thickening and calcification may lead to valvular stenosis or regurgitation [4,5,6,7,8,9,10,11,12,13,14,15]. GAG accumulation in the spongiosa of cardiac valves, myointima of coronary arteries, and myocardium has been associated with cardiomyopathy and valve defects [16]. The onset and extent of cardiovascular involvement is different in each MPS type, and defects in the cardiac structures in association with cardiac dysfunction meaningfully increase morbidity and mortality in these patients [17,18,19,20].

Assessing myocardial strain using two-dimensional speckle-tracking echocardiography (2D STE) has been reported to be a reliable tool for identifying nascent myocardial injury, which is imperative in the early detection of subclinical cardiac dysfunction and prognosis of several cardiac diseases [21,22]. Longitudinal left ventricular (LV) function is a sensitive and well-documented clinical marker of early stage LV systolic dysfunction in subjects with ventricular hypertrophy, hypertensive remodeling, diabetic cardiomyopathy, and heart failure with preserved ejection fraction [23,24,25,26]. Consequently, using myocardial deformation imaging in clinical practice may be of value in the early diagnosis of myocardial dysfunction. However, STE has not yet been broadly used to evaluate myocardial mechanics in patients with MPS [21,27,28]. In this study, we aimed to determine the cardiac structure and function of Taiwanese patients with various MPS types using 2D STE and conventional echocardiography.

## 2. Materials and Methods

### 2.1. Study Population

Cardiac features of 53 patients with MPS (31 men and 22 women; median age of 16.0 years; age range 1.1–34.9 years) were evaluated using 2D STE and conventional echocardiography from December 2013 to December 2018 at MacKay Memorial Hospital. The diagnosis of MPS was confirmed by measuring the enzymatic activities of particular lysosomal hydrolases in leukocytes or skin fibroblasts using two-dimensional electrophoresis of the urinary GAGs and/or through mutational analysis [29,30]. Patients with MPS I were further subdivided into those with Hurler, H/S, or Scheie syndrome based on their clinical manifestations and mutation analysis. There were three patients with MPS I (H/S), four with MPS I (Scheie), 16 with MPS II, nine with MPS III, 14 with MPS IVA, and seven with MPS VI in our cohort. No patient had received hematopoietic stem cell transplantation. Twenty-two patients (one with MPS I, eight with MPS II, seven with MPS IVA, and six with MPS VI) were under enzyme replacement therapy (ERT) at the time of echocardiographic evaluation. Patients with MPS I received 0.58 mg/kg/week intravenous laronidase (Aldurazyme^®^, Genzyme Corporation, Cambridge, MA, USA), those with MPS II received 0.5 mg/kg/week intravenous idursulfase (Elaprase^®^, Shire Pharmaceutical Inc., Lexington, MA, USA), those with MPS IVA received 2.0 mg/kg/week intravenous elosulfase alfa (Vimizim^®^, BioMarin Pharmaceutical Inc., San Rafael, CA, USA), and those with MPS VI received 1.0 mg/kg/week intravenous galsulfase (Naglazyme^®^, BioMarin Pharmaceutical Inc., San Rafael, CA, USA). Written informed consent for cardiac evaluations was obtained from a parent if the patient was aged <18 years. All procedures followed were in accordance with the ethical standards of the responsible committee on human experimentation (institutional and national) and with the Declaration of Helsinki of 1975, as revised in the year 2000. The Institutional Review Board of MacKay Memorial Hospital approved this study (14MMHIS281, approval date: 30 March 2015), and written informed consent was obtained from all of the patients or their parents who were included in the study

### 2.2. Measurements of Echocardiographic Parameters

#### 2.2.1. 2D STE

We performed LV deformational imaging using baseline 2D STE from three LV apical views for longitudinal strain (EchoPAC version 10.8; GE Vingmed Ultrasound AS, Horten, Norway), as described in our previous work [31]. We manually traced the endomyocardial border with epicardial tracing automatically generated by algorithm, with minor adjustment to cover the whole myocardial wall thickness. Based on automated speckle-tracking algorithms, LV global longitudinal strain (GLS) was then averaged from the three individual LV apical views (two-, four-, and three-chamber views, respectively). The average frame rate in current analysis was 78.1 ± 3.1 frames/sec. Subsequently, these values were compared with the normal values based on a study by Marcus et al. [32].

#### 2.2.2. Conventional Echocardiography

Echocardiography was performed using a Vividi system (GE Vingmed Ultrasound, Horten, Norway) equipped with a 2- to 4-MHz transducer (3S-RS) during the recruitment period of this study. Data were digitally stored and analyzed by one experienced cardiologist (CLH) to minimize any potential interobserver variations as described in our previous study [31]. The left ventricular (LV) diastolic and systolic diameters were measured by the M-mode. The systolic function of the LV was evaluated based on the ejection fraction according to the Simpson method. For children, an ejection fraction of <50% was defined as abnormal, whereas for adults, an ejection fraction of <52% for men and <54% for women was defined as abnormal [33]. A shortening fraction of <28% was considered abnormal in both adults and children. Diastolic filling was assessed using the E/A ratio by measuring the mitral-inflow according to the pattern-peak early filling (E) and late filling (A) velocities. Moreover, the systolic function was assessed according to the ejection and shortening fractions [34]. Existence of diastolic dysfunction was indicated by a reversed E/A ratio (E/A ratio <1). The severity of valvular stenosis and regurgitation was assessed and graded as follows: 0 (none), 1 (mild), 2 (moderate), and 3 (severe), based on the European Society of Cardiology guidelines [12,13,14,15,35,36]: mild aortic stenosis (AS) = a valve area > 1.5 cm^2^ and a mean gradient < 30 mmHg; moderate AS = a valve area of 1.0–1.5 cm^2^ and a mean gradient of 30–50 mmHg; severe AS = a valve area < 1.0 cm^2^ and a mean gradient > 50 mmHg; mild mitral stenosis (MS) = a valve area > 1.5 cm^2^ and a mean gradient < 5 mmHg; moderate MS = a valve area between 1.0–1.5 cm^2^ and a mean gradient between 5–10 mmHg; and severe MS = a valve area < 1.0 cm^2^ and a mean gradient >10 mmHg. Because the frequency of physiological tricuspid regurgitation is high in the general population, we did not include tricuspid regurgitation as a pathological finding in this study.

We recorded data on left ventricular mass index (LVMI), interventricular septal end-diastolic dimension (IVSd), left ventricular posterior wall end-diastolic dimension (LVPWd), left ventricular end-diastolic dimension (LVIDd), and aortic diameter obtained by echocardiographic evaluations. The relative wall thickness (RWT) was calculated as (2×LVPWd)/LVIDd. Three patterns of LV remodeling were determined based on measurements of the LVMI and RWT, namely, concentric remodeling (normal LVMI and RWT > 0.42), eccentric hypertrophy (LVMI *z* score >2 and RWT <= 0.42), and concentric hypertrophy (LVMI *z* score >2 and RWT >0.42) [37]. Measurements of the aorta were made on the sinus by leading-edge-to-leading-edge. The LVMI was calculated using the Devereux formula and indexed by the height *z* score with normal values according to the report by Foster et al. [38]. These values were compared with normal values according to the study by Kampmann et al. [39].

All echocardiographic parameters were transformed into a *z* score derived by subtracting the mean reference value from an individual observed value, then dividing the difference by the standard deviation from the reference value. A *z* score value between −2 and +2 was considered normal.

### 2.3. Data Analysis and Statistics

Sex, MPS type, age, height, weight, and the duration of receiving ERT at the time of echocardiographic assessments were recorded for each patient. Descriptive statistics, including the mean and standard deviation, of all echocardiographic parameters were calculated. The relationships between the age and various echocardiographic parameters were established using a Pearson’s correlation coefficient (*r*), and the significance was determined using the Fisher’s *r–z* transformations to obtain two-tailed *p*-values. All statistical analyses were conducted using SPSS version 11.5 (SPSS Inc., Chicago, IL, USA), and any differences with a *p* < 0.05 were considered statistically significant.

## 3. Results

**Figure 1:**
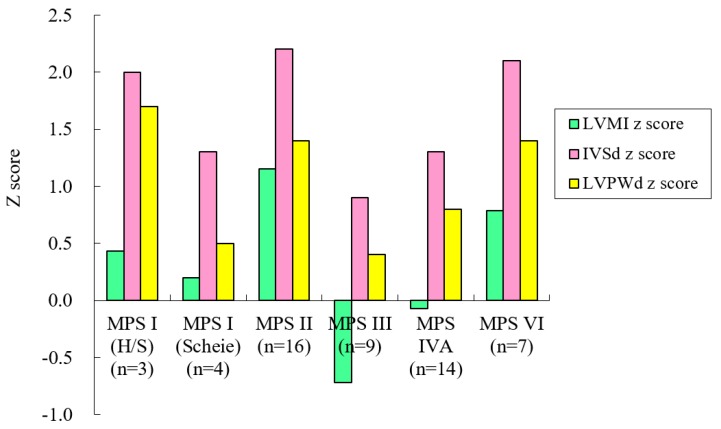
The *z* scores of LVMI, IVSd, and LVPWd by MPS type in the 53 patients. MPS, mucopolysaccharidosis; LVMI, left ventricular mass index; IVSd, interventricular septum thickness in diastole; LVPWd, left ventricular posterior wall thickness in diastole; H/S: Hurler-Scheie.

**Figure 2:**
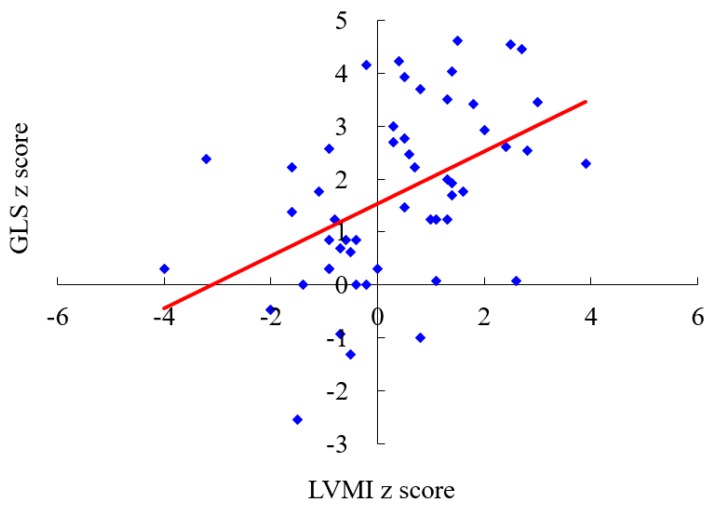
The LVMI *z* score is positively correlated with the GLS *z* score (*n* = 53, *r* = 0.469, *p* < 0.01). LVMI, left ventricular mass index; GLS, global longitudinal strain. The red line represents the trendline.

**Figure 3:**
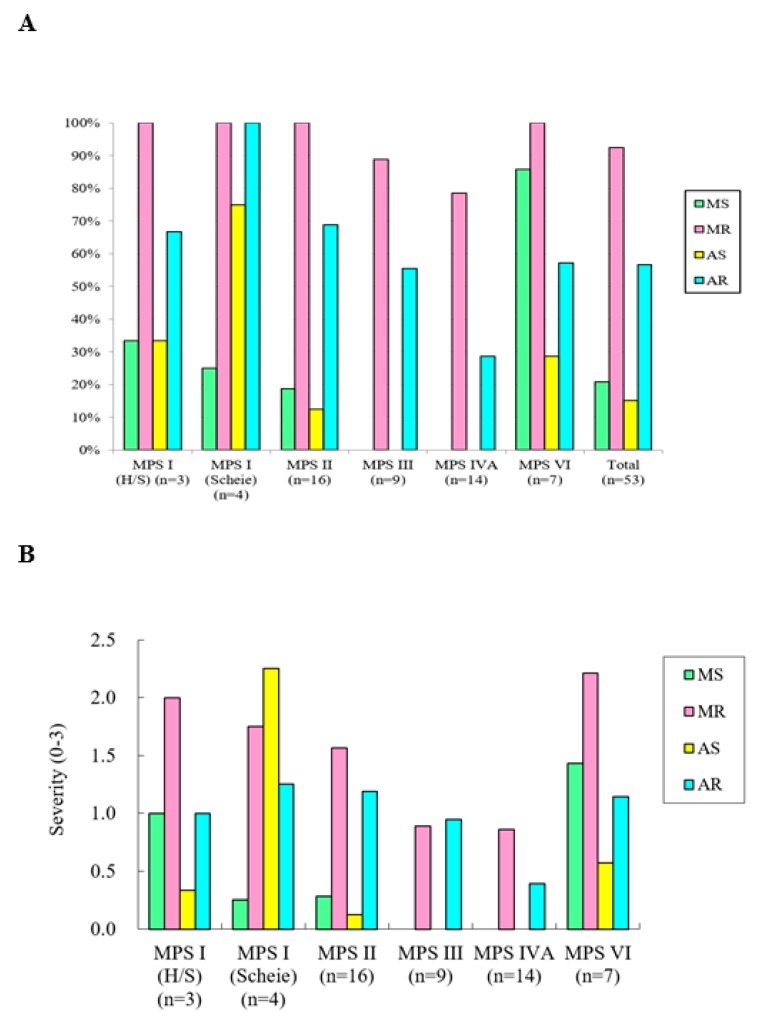
(**A**) Incidence of valvular heart disease by MPS type in the 53 patients. (**B**) Mean severity scores of valvular heart disease by MPS type in the 53 patients. Severity of valvular stenosis and regurgitation (MS, MR, AS, AR) was estimated and graded as follows: 0 (none), 1 (mild), 2 (moderate), and 3 (severe). MPS, mucopolysaccharidosis; MS, mitral stenosis; MR, mitral regurgitation; AS, aortic stenosis; AR, aortic regurgitation; H/S: Hurler-Scheie.

**Figure 4:**
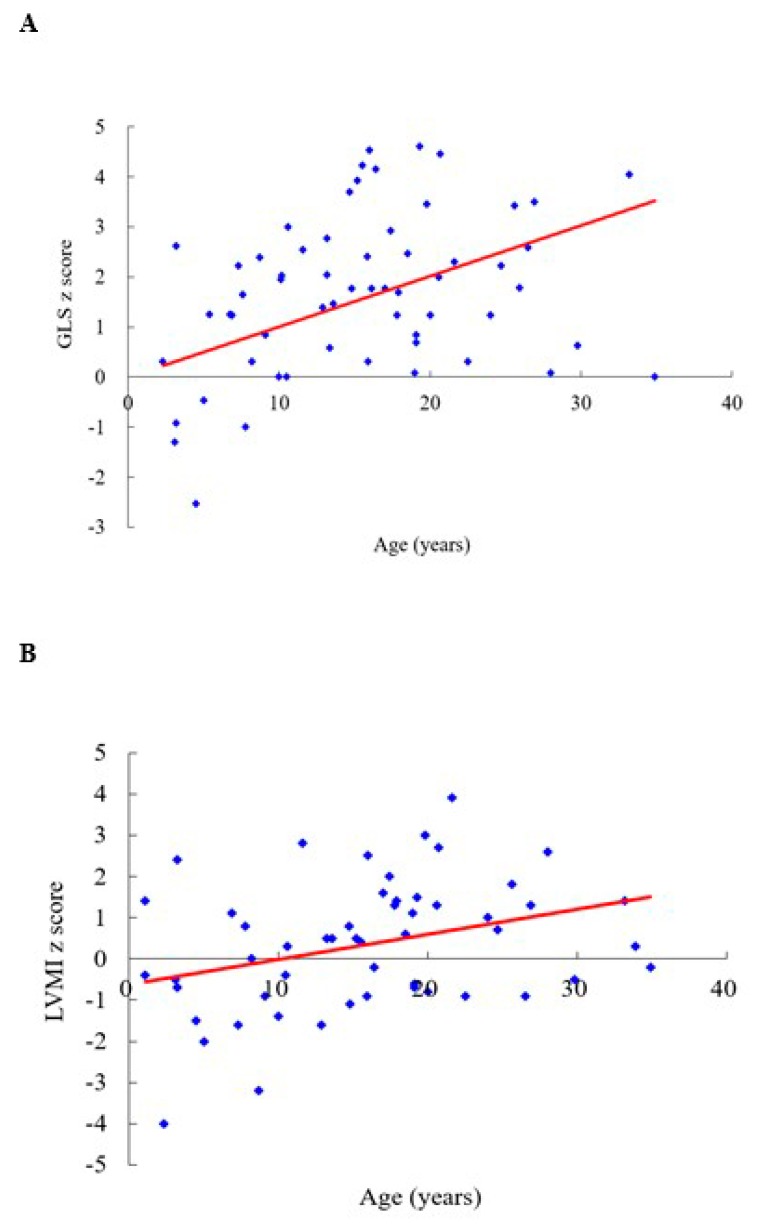
(**A**) The GLS z score is positively correlated with age (n = 53, *r* = 0.323, *p* < 0.05). (**B**) The LVMI *z* score is positively correlated with age (n = 53, *r* = 0.335, *p* < 0.05). GLS, global longitudinal strain; LVMI, left ventricular mass index. The red line represents the trendline.

**Figure 5:**
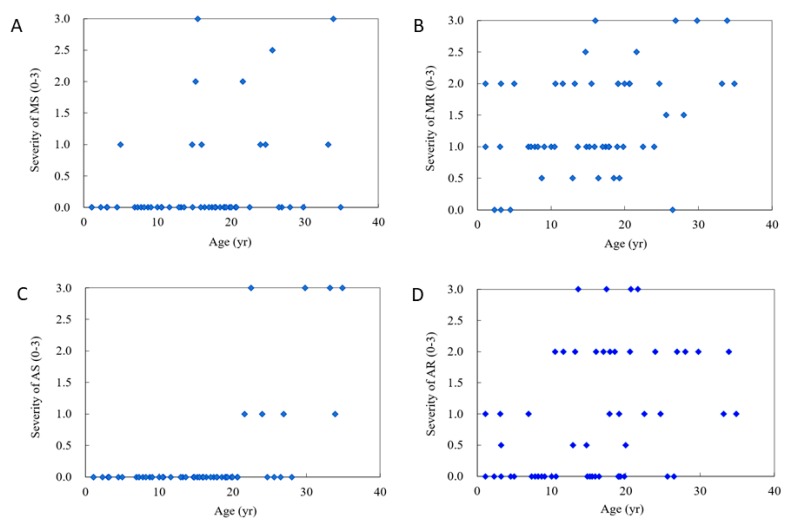
The relationships between age and severity of cardiac valve abnormalities in the 53 patients with mucopolysaccharidosis (severity score: 3: severe, 2: moderate, 1: mild, 0: normal). (**A**) MS, mitral stenosis (*r* = 0.314, *p* < 0.05); (**B**) MR, mitral regurgitation (*r* = 0.410, *p* < 0.01); (**C**) AS, aortic stenosis (*r* = 0.579, *p* < 0.01); (**D**) AR, aortic regurgitation (*r* = 0.371, *p* < 0.01). The blue dot represents the value of age and severity of cardiac valve abnormalities in the 53 patients.

**Figure 6:**
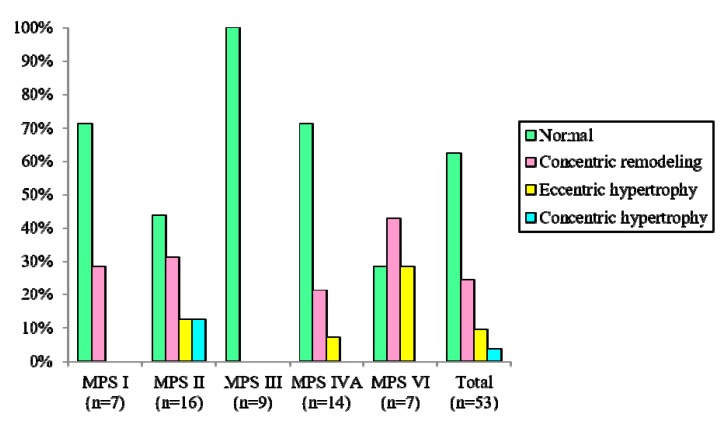
Percentages of left ventricular remodeling patterns in various MPS types. MPS, mucopolysaccharidosis.

Table 1 shows clinical and echocardiographic features of the 53 patients with different MPS types. The mean *z* scores of the GLS, LVMI, IVSd, LVPWd, and aortic diameter in all 53 patients with MPS were 1.71, 0.35, 1.66, 1.03, and 3.15, respectively. *Z* scores of >2 were identified in 45%, 13%, 40%, 13%, and 70% of the GLS, LVMI, IVSd, LVPWd, and aortic diameter, respectively. The greatest deviation from the reference values of GLS was observed in the patients with MPS VI, followed by those with MPS II and MPS I. Patients with MPS II had the largest LVMI and IVSd, followed by those with MPS VI. Patients with MPS IVA had the largest aortic diameter, followed by those with MPS II (Table 2 and Figure 1). The GLS *z* score was positively correlated with the LVMI *z* score (*p* < 0.01) (Figure 2). Diastolic dysfunction (reversed ratio between early and late (atrial) ventricular filling velocity (E/A ratio < 1)) was identified in 12 patients (23%). Ejection and shortening fractions were abnormal in four (8%) and seven (13%) patients, respectively. Forty-nine (92%) patients with MPS had different types and degrees of severity of valvular heart disease. Mitral regurgitation (MR) (92%) was the most common presentation, followed by aortic regurgitation (AR) (57%), MS (21%), and AS (15%). All patients with MPS I, II, and VI had valvular heart disease, compared to 89% of those with MPS III and 79% of those with MPS IV. Patients with MPS VI had the highest severity of MS and MR, whereas those with MPS I (Scheie) had the highest severity of AS and AR (Table 3 and Figure 3). The *z* scores of GLS and LVMI and severity scores of MS, MR, AS, and AR were all positively correlated with increasing age (*p* < 0.05) (Figure 4 and Figure 5). Twenty patients (38%) had an LV remodeling pattern, including 13 (25%) with LV concentric remodeling, five (9%) with eccentric hypertrophy, and two (4%) with concentric hypertrophy. The remaining 33 patients (62%) had normal LV geometry. Among the various MPS types, most patients with MPS VI (71%) had LV remodeling, followed by those with MPS II (56%). However, no patient with MPS III had LV remodeling (Table 4 and Figure 6).

## 4. Discussion

To the best of our knowledge, this is the first study to delineate the cardiac structure and function of Asian patients with various MPS types in a single population using 2D STE and conventional echocardiography and compare the results with normal values, including young adults, based on the reports of Marcus et al. [32] and Kampmann et al. [39]. In this study, we evaluated the LV contractile function according to myocardial deformation imaging using a 2D STE technique in patients with different MPS types and ages. We found that the ejection and shortening fractions were abnormal in 8% and 13% of patients with MPS, respectively. However, a GLS *z* score of >2 was observed in 45% of these patients. The findings of a GLS *z* score of >2 in the absence of LV ejection and shortening fraction abnormalities suggests that 2D STE is an early marker for LV dysfunction in patients with MPS. We demonstrated early stage systolic dysfunction using the more sensitive LV deformation parameter, GLS *z* score, which preceded global LV systolic dysfunction as traditionally assessed using LV ejection and shortening fractions. Additionally, we observed that the GLS *z* score as a measure of myocardial deformation was significantly associated with the LVMI *z* score and age. In addition, diastolic dysfunction (E/A ratio < 1) was identified in 12 patients (23%). MPS was related to myocardial deformation and more impaired diastolic function and a decline in the global LV systolic mechanics, despite globally preserved LV ejection and shortening fractions. These results were consistent with those of previous studies [21,27,28].

Conventional two-dimensional echocardiography is a clinically useful tool, however its use in determining the early stages of myocardial dysfunction is limited. In recent years, 2D STE imaging has been used to independently identify early stage LV dysfunction before conventional echo parameters become abnormal. Conventional echocardiography can detect overt global systolic dysfunction with regard to the reduced LV ejection fraction in patients with MPS; however, it is not sufficiently sensitive to identify early stage LV dysfunction. Diastolic function parameters may be useful; however, they are only indirect measures of LV contractile function and are considerably affected by hemodynamic load conditions. Accordingly, STE may help in the identification of early stage LV systolic dysfunction in patients with MPS before the development of obvious heart failure and is more reliable and practicable [26].

All patients with MPS in the present study had cardiac abnormalities. Overall, 40% of these patients had an IVSd *z* score of >2, whereas only 13% had an LVMI *z* score of >2. IVSd is related to the LVMI, and the clinical significance of an isolated increase in IVSd may be caused by a LV remodeling pattern in these patients. Our results revealed that the most significant LV myocardial deformation, hypertrophy, and valvular heart disease were observed in patients with MPS VI, II, and I, followed by those with MPS IV, and that patients with MPS III had the mildest manifestations. Deformed mitral or aortic valves with varying degrees of severity were commonly noted (92%) in our patients with MPS. In this cohort, mitral valve abnormalities (92%) were more prevalent than aortic valve abnormalities (57%), and valvular regurgitation (92%) was more common than valvular stenosis (28%), which is consistent with previous studies [4,5,6,7,8]. The most prevalent cardiac valve abnormality was MR (92%), followed by AR (57%), MS (21%), and AS (15%). Consistent with the report by Mohan et al. [9], valvular stenosis and regurgitation in our patients with MPS worsened with increasing age, which is in accordance with the progressive nature of this disease. Among the various MPS types, all patients with MPS I, II, and VI had valvular heart disease, compared to 89% of those with MPS III and 79% of those with MPS IV. Patients with MPS VI had the highest severity of MS and MR, whereas those with MPS I (Scheie) had the highest severity of AS and AR. Previous studies have reported that abnormal catabolism of dermatan sulfate in patients with MPS I, II and VI leads to dermatan-sulfate GAG accumulation in the cardiac valves, resulting in valvular thickening and other cardiac defects [7,8]. As valvular heart diseases have been shown to contribute to worsened global ventricular deformations, we speculated that more impaired GLS in MPS VI, II, and I may be partly attributable to the accompanied valvular lesions [40]. The major storage products of MPS III and MPS IV are heparan sulfate and keratan sulfate, respectively. Therefore, cardiac lesions may be less prominent in patients with MPS III and IV than in those with MPS I, II, and VI [14,15,41].

Aortic root dilatation classically evolves through cystic medial degeneration and migration of the smooth muscle cells from the tunica media to the tunica intima with age [42]. Bolourchi et al. [43] reported that aortic root dilatation was highly prevalent in all patients with MPS (35%) and that those with MPS IVA had the highest prevalence of aortic root dilatation (62.5%), which is consistent with our results (aortic diameter *z* score >2 in 70% of patients with all MPS types and 93% of those with MPS IVA). Routine screening for this potentially critical observation should be integrated into the multidisciplinary care of patients with MPS.

Ventricular remodeling leads to alterations in the ventricular architecture with associated increases in volume and altered chamber configuration, thereby causing myocyte hypertrophy and apoptosis, myofibroblast proliferation, and interstitial fibrosis [44]. Few reports have described the LV remodeling pattern in patients with MPS. In our cohort, 38% of patients with MPS had LV remodeling pattern, including 25% with LV concentric remodeling, 9% with eccentric hypertrophy, and 4% with concentric hypertrophy. Among all MPS types, most patients with MPS VI (71%) had LV remodeling, followed by those with MPS II (56%), MPS IVA (29%), and MPS I (29%). Each of these patterns was associated with a higher risk of subsequent cardiovascular events than a normal LV morphology, with each of these three patterns carrying a progressively worse prognosis [44].

The ERT for some MPS disorders appears to be effective in stabilizing or reducing cardiac hypertrophy, and better results may be associated with initiating the ERT at a younger age. There is currently no definitive evidence of the effects of ERT on valvulopathy [10,13,14]. However, due to the progressive nature of MPS, starting the ERT before the occurrence of irreversible cardiac damage may contribute to a better clinical outcome. As such, it is essential to make an early diagnosis via screening programs for high-risk populations or newborns [45,46,47,48].

## 5. Limitations

In this study, there was no healthy control group to compare the echocardiographic parameters with those of our patients. Further, not all patients in our study cohort had echocardiographic data before the ERT. Consequently, we used reference values from a Caucasian population due to the lack of data from an Asian population, under the recognition that longitudinal strain values from single four-chamber may be highly consistent from those averaged from three planes based on the systemic review [49,50]. The small sample size of patients with each type of MPS reflects the rare nature of this genetic disorder. Additionally, both the age range (1.1–34.9 years) and degree of disease severity differed widely. Therefore, further studies with larger cohorts and longer follow-up periods are warranted to validate our findings.

## 6. Conclusions

Our results showed that a substantial proportion of patients with MPS had myocardial deformation, cardiac hypertrophy, aortic dilatation, and valvular heart disease. Assessing the LV function using 2D STE to evaluate myocardial deformation may help to stratify the risk of MPS. STE was more sensitive than global LV ejection fraction and fractional shortening, and its derived parameter, GLS, may prove especially useful in patients with subclinical LV dysfunction prior to the development of obvious systolic dysfunction. Cardiac abnormalities in our patients worsened with increasing age in accordance with the progressive nature of this disease. The most significant LV myocardial deformation, hypertrophy and valvular heart disease, were observed in patients with MPS VI, II, and I, followed by those with MPS IV, whereas those with the MPS III had the mildest manifestations. Despite the globally preserved LV ejection and shortening fractions, MPS was associated with myocardial deformation, more impaired diastolic function, and decline in the global LV systolic mechanics.

## Figures and Tables

**Table 1:** Clinical and echocardiographic features of the 53 patients with MPS.

No.	Gender	MPS Type	Age (Years)	ERT Duration (Years)	GLS z Score	LVMI z Score	IVSd z Score	LVPWd z Score	AoD z Score	EF (%)	SF (%)	E/A Ratio	RWT	Left Ventricular Remodeling Pattern	MS	MR	AS	AR
1	F	I (H/S)	1.1		1.9	1.4	**2.9**	**2.1**	−0.1	54	**27**	3.22	0.413	Normal geometry	0	2	0	1
2	F	I (H/S)	1.1		0.8	−0.4	**2.3**	1.7	**3.6**	71	35	1.44	**0.449**	Concentric remodeling	0	1	0	0
3	F	I (H/S)	33.9		**2.7**	0.3	1.0	1.1	**4.4**	62	33	**0.94**	0.337	Normal geometry	3	3	1	2
4	M	I (Scheie)	13.2		**2.8**	0.5	1.1	0.7	0.4	71	40	2.56	0.346	Normal geometry	0	2	0	2
5	F	I (Scheie)	22.5		0.3	−0.9	1.7	0.2	−0.4	71	40	1.71	**0.423**	Concentric remodeling	0	1	3	1
6	M	I (Scheie)	33.2		**4.0**	1.4	1.7	0.7	1.4	66	37	1.16	0.376	Normal geometry	1	2	3	1
7	M	I (Scheie)	34.9		0.0	−0.2	0.7	0.4	0.9	60	32	**0.91**	0.380	Normal geometry	0	2	3	1
8	M	II (mild)	6.9		1.2	1.1	**3.2**	1.5	**3.3**	74	42	1.05	**0.434**	Concentric remodeling	0	1	0	1
9	M	II (mild)	7.3		**2.2**	−1.6	0.8	−0.2	**7.0**	68	37	1.44	0.348	Normal geometry	0	1	0	0
10	M	II (mild)	10.0	5.0	0.0	−1.4	−0.9	−0.1	**2.9**	71	39	1.73	0.335	Normal geometry	0	1	0	0
11	M	II (mild)	10.6	6.5	**3.0**	0.3	**2.4**	1.4	2.0	62	32	1.13	**0.455**	Concentric remodeling	0	2	0	0
12	M	II (mild)	11.6	5.7	**2.5**	**2.8**	1.6	1.7	**4.2**	**41**	**20**	**0.89**	0.351	Eccentric hypertrophy	0	2	0	2
13	M	II (mild)	17.4	2.4	**2.9**	2	**2.3**	1.2	**5.3**	73	42	2.61	0.349	Normal geometry	0	1	0	3
14	M	II (mild)	19.8		**3.5**	3	**3.7**	**2.4**	**4.2**	74	43	2.92	**0.421**	Concentric hypertrophy	0	1	0	0
15	M	II (mild)	20.6	7.4	**2.0**	1.3	0.5	0.3	0.7	**50**	**26**	1.36	0.309	Normal geometry	0	2	0	2
16	M	II (mild)	20.7	2.4	**4.5**	**2.7**	**2.6**	1.9	**4.7**	**51**	**26**	1.45	0.381	Eccentric hypertrophy	0	2	0	3
17	M	II (mild)	24.0	6.5	1.2	1	1.9	1.4	**3.7**	62	33	**0.85**	0.391	Normal geometry	1	1	1	2
18	M	II (mild)	25.6	3.5	**3.4**	1.8	**2.7**	1.7	1.3	73	41	**0.67**	**0.451**	Concentric remodeling	2.5	1.5	0	0
19	M	II (severe)	3.1		−1.3	−0.5	0.7	0.9	**3.8**	63	33	1.17	0.359	Normal geometry	0	1	0	1
20	M	II (severe)	3.2		**2.6**	**2.4**	**4.3**	**2.5**	**3.8**	63	33	1.48	**0.421**	Concentric hypertrophy	0	2	0	0.5
21	M	II (severe)	14.7		**3.7**	0.8	**4.1**	**2.1**	**2.4**	76	44	**0.95**	**0.4204**	Concentric remodeling	1	2.5	0	0.5
22	M	II (severe)	17.9		1.7	1.4	1.4	1.5	**2.6**	76	44	1.42	0.382	Normal geometry	0	1	0	2
23	M	II (severe)	26.9		**3.5**	1.3	**3.9**	**2.1**	**6.0**	65	35	1.14	**0.444**	Concentric remodeling	0	3	1	2
24	F	IIIA	8.7		**2.4**	−**3.2**	0.0	−0.6	0.9	52	**26**	1.31	0.301	Normal geometry	0	0.5	0	0
25	F	IIIB	10.5		0.0	−0.4	1.4	0.7	1.8	71	40	1.46	0.366	Normal geometry	0	1	0	2
26	F	IIIB	12.9		1.4	−1.6	0.1	0.4	1.3	75	43	1.06	0.391	Normal geometry	0	0.5	0	0.5
27	M	IIIA	13.6		1.5	0.5	0.9	0.4	1.7	73	42	1.26	0.338	Normal geometry	0	1	0	3
28	M	IIIA	16.4		**4.2**	−0.2	2.0	0.9	**5.2**	68	38	1.37	0.382	Normal geometry	0	0.5	0	0
29	F	IIIB	18.5		**2.5**	0.6	**2.4**	1.5	**2.4**	60	32	**0.88**	0.345	Normal geometry	0	0.5	0	2
30	F	IIIB	19.1		0.7	−0.7	1.0	0.1	**2.6**	69	38	**0.89**	0.349	Normal geometry	0	2	0	0
31	F	IIIB	19.1		0.8	−0.6	0.2	0.8	**3.8**	72	41	**0.93**	0.382	Normal geometry	0	2	0	1
32	M	IIIC	26.5		**2.6**	−0.9	0.5	−0.1	**2.7**	56	29	1.08	0.333	Normal geometry	0	0	0	0
33	M	IVA	2.3		0.3	− **4**	0.4	0.5	**2.5**	62	31	1.11	**0.476**	Concentric remodeling	0	0	0	0
34	M	IVA	3.2	1.7	−0.9	−0.7	0.4	0.3	**2.5**	72	40	1.73	0.312	Normal geometry	0	0	0	0
35	M	IVA	4.5	1.7	−**2.5**	−1.5	0.1	0.5	**2.5**	81	49	1.24	0.347	Normal geometry	0	0	0	0
36	F	IVA	7.8		−1.0	0.8	**2.3**	1.9	1.0	56	28	1.10	0.394	Normal geometry	0	1	0	0
37	F	IVA	8.2		0.3	0	**3.5**	1.6	**3.7**	71	39	1.16	**0.429**	Concentric remodeling	0	1	0	0
38	M	IVA	9.1	1.8	0.8	−0.9	0.0	−0.2	**3.9**	61	31	1.40	0.297	Normal geometry	0	1	0	0
39	F	IVA	14.8		1.8	−1.1	1.3	0.6	**2.6**	72	40	1.82	0.373	Normal geometry	0	1	0	0
40	M	IVA	15.9	2.0	0.3	−0.9	1.2	0.8	**8.4**	70	38	1.42	0.375	Normal geometry	0	1	0	0
41	F	IVA	17.0	2.2	1.8	1.6	0.7	0.1	**5.3**	76	45	1.42	0.255	Normal geometry	0	1	0	2
42	F	IVA	17.8		1.2	1.3	**2.9**	**2.7**	**4.9**	68	37	1.19	**0.448**	Concentric remodeling	0	1	0	1
43	F	IVA	19.0	2.2	0.1	1.1	1.3	0.5	**5.3**	70	39	1.06	0.349	Normal geometry	0	1	0	0
44	F	IVA	19.3		**4.6**	1.5	1.2	0.5	**4.5**	67	36	1.29	0.312	Normal geometry	0	0.5	0	0
45	F	IVA	20.0		1.2	−0.8	0.5	0.1	**6.8**	78	45	1.06	0.323	Normal geometry	0	2	0	0.5
46	M	IVA	28.0	2.3	0.1	**2.6**	**2.1**	1.0	**6.3**	53	**26**	1.06	0.324	Eccentric hypertrophy	0	1.5	0	2
47	M	VI	5.0		−0.5	−2	0.1	0.0	1.0	65	34	**0.91**	0.321	Normal geometry	1	2	0	0
48	M	VI	15.2	7.7	**3.9**	0.5	**3.3**	**3.2**	**3.8**	72	39	9.40	**0.510**	Concentric remodeling	2	1	0	0
49	F	VI	15.5	1.5	**4.2**	0.4	**2.8**	1.2	1.2	75	43	**0.81**	**0.424**	Concentric remodeling	3	2	0	0
50	M	VI	16.0	7.7	**4.5**	**2.5**	**2.3**	1.5	**4.6**	76	44	1.11	0.371	Eccentric hypertrophy	1	3	0	2
51	F	VI	21.6	9.9	**2.3**	**3.9**	**2.2**	1.5	**2.6**	**50**	**25**	**0.83**	0.323	Eccentric hypertrophy	2	2.5	1	3
52	F	VI	24.7	8.0	**2.2**	0.7	**2.1**	1.5	**3.0**	72	40	1.35	**0.463**	Concentric remodeling	1	2	0	1
53	F	VI	29.8	3.3	0.6	−0.5	2.0	1.1	0.2	76	44	1.22	0.379	Normal geometry	0	3	3	2

MPS, mucopolysaccharidosis; ERT, enzyme replacement therapy; GLS, global longitudinal strain; LVMI, left ventricular mass index; IVSd, interventricular septal end-diastolic dimension; LVPWd, left ventricular posterior wall end-diastolic dimension; AoD, aortic diameter; EF, ejection fraction; SF, shortening fraction; E/A: ratio between early and late (atrial) ventricular filling velocity; RWT, relative wall thickness; MS, mitral stenosis; MR, mitral regurgitation; AS, aortic stenosis; AR, aortic regurgitation; H/S: Hurler-Scheie. Severity of valvular stenosis and regurgitation (MS, MR, AS, AR) were estimated and graded on the following scores: 0 (none), 1 (mild), 2 (moderate), and 3 (severe). The abnormal values are presented in boldface, which include: *z* score >2 or <−2; EF: for children < 50%; for adults < 52% for men and < 54% for women; SF < 28%; E/A ratio < 1; RWT > 0.420.

**Table 2:** The *z* scores of GLS, LVMI, IVSd, LVPWd, and aortic diameter by MPS type in the 53 patients. Values are displayed as mean (standard deviation).

MPS Type	*n*	Gender (M/F)	Age (Years)	Age Range (Years)	GLS z Score	LVMI z Score	IVSd z Score	LVPWd z Score	AoD z Score
MPS I (H/S)	3	0/3	12.0 (18.9)	1.1–33.9	1.82 (0.93)	0.43 (0.91)	2.04 (0.95)	1.67 (0.50)	2.62 (2.38)
MPS I (Scheie)	4	3/1	26.0 (10.1)	13.2–34.9	1.78 (1.95)	0.20 (0.98)	1.31 (0.47)	0.51 (0.25)	1.69 (1.66)
MPS II	16	16/0	15.0 (7.7)	6.9–26.9	2.29 (1.48)	1.15 (1.39)	2.20 (1.47)	1.39 (0.81)	3.61 (1.66)
MPS III	9	3/6	16.1 (5.4)	8.7–26.5	1.77 (1.25)	−0.72 (1.15)	0.95 (0.86)	0.45 (0.64)	2.49 (1.33)
MPS IVA	14	6/8	13.3 (7.6)	2.3–28.0	0.58 (1.65)	−0.07 (1.69)	1.29 (1.06)	0.79 (0.78)	4.30 (2.02)
MPS VI	7	3/4	18.3 (8.0)	5.0–29.8	2.48 (1.90)	0.79 (1.93)	2.13 (0.99)	1.45 (0.95)	2.35 (1.59)
Total	53	31/22	15.3 (8.2)	1.1–34.9	1.71 (1.66)	0.35 (1.57)	1.66 (1.20)	1.03 (0.84)	3.15 (1.93)

MPS, mucopolysaccharidosis; M/F, male/female; GLS, global longitudinal strain; LVMI, left ventricular mass index; IVSd, interventricular septum thickness in diastole; LVPWd, left ventricular posterior wall thickness in diastole; AoD, aortic diameter; H/S: Hurler-Scheie.

**Table 3:** Mean severity scores of valvular heart disease by MPS type in the 53 patients. 0, normal; 1, mild; 2, moderate; 3, severe. Values are displayed as mean (standard deviation).

MPS Type	*n*	Gender (M/F)	Age (Years)	MS	MR	AS	AR
MPS I (H/S)	3	0/3	12.0 (18.9)	1.00	2.00	0.33	1.00
MPS I (Scheie)	4	3/1	26.0 (10.1)	0.25	1.75	2.25	1.25
MPS II	16	16/0	15.0 (7.7)	0.28	1.56	0.13	1.19
MPS III	9	3/6	16.1 (5.4)	0.00	0.89	0.00	0.94
MPS IVA	14	6/8	13.3 (7.6)	0.00	0.86	0.00	0.39
MPS VI	7	3/4	18.3 (8.0)	1.43	2.21	0.57	1.14
Total	53	31/22	15.3 (8.2)	0.35	1.39	0.30	0.92

MPS, mucopolysaccharidosis; M/F, male/female; MS, mitral stenosis; MR, mitral regurgitation; AS, aortic stenosis; AR, aortic regurgitation; H/S: Hurler-Scheie.

**Table 4:** Number and percentages of left ventricular remodeling patterns in various MPS types.

Left Ventricular Remodeling Pattern	Normal	Concentric Remodeling	Eccentric Hypertrophy	Concentric Hypertrophy
MPS I (*n* = 7)	5 (71%)	2 (29%)	0	0
MPS II (*n* =16)	7 (44%)	5 (31%)	2 (13%)	2 (13%)
MPS III (*n* = 9)	9 (100%)	0	0	0
MPS IVA (*n* = 14)	10 (71%)	3 (21%)	1 (7%)	0
MPS VI (*n* = 7)	2 (29%)	3 (43%)	2 (29%)	0
Total (*n* = 53)	33 (62%)	13 (25%)	5 (9%)	2 (4%)

MPS, mucopolysaccharidosis.

## Abbreviations

MPS	Mucopolysaccharidosis
GAGs	Glycosaminoglycans
H/S	Hurler-Scheie
2D STE	Two-dimensional speckle-tracking echocardiography
LV	Left ventricular
ERT	Enzyme replacement therapy
GLS	Global longitudinal strain
E/A	Ratio between early and late (atrial) ventricular filling velocity
AS	Aortic stenosis
MS	Mitral stenosis
LVMI	Left ventricular mass index
IVSd	Interventricular septal end-diastolic dimension
LVPWd	Left ventricular posterior wall end-diastolic dimension
LVIDd	Left ventricular end-diastolic dimension
RWT	Relative wall thickness
